# The PS4-likelihood ratio calculator: flexible allocation of evidence weighting for case-control data in variant classification

**DOI:** 10.1136/jmg-2024-110034

**Published:** 2024-09-03

**Authors:** Charlie F Rowlands, Alice Garrett, Sophie Allen, Miranda Durkie, George J Burghel, Rachel Robinson, Alison Callaway, Joanne Field, Bethan Frugtniet, Sheila Palmer-Smith, Jonathan Grant, Judith Pagan, Trudi McDevitt, Terri P McVeigh, Helen Hanson, Nicola Whiffin, Michael Jones, Clare Turnbull, CanVIG-UK, C Turnbull, A Garrett, L Loong, S Choi, B Torr, S Allen, M Durkie, A Callaway, J Drummond, GJ Burghel, R Robinson, IR Berry, AJ Wallace, DM Eccles, M Tischkowitz, S Ellard, H Hanson, E Baple, DG Evans, E Woodward, F Lalloo, S Samant, A Lucassen, A Znaczko, A Shaw, A Ansari, A Kumar, A Donaldson, A Murray, A Ross, A Taylor-Beadling, A Taylor, A Innes, AF Brady, A Kulkarni, AC Hogg, A Ramsay Bowden, A Hadonou, B Coad, B McIldowie, B Speight, B DeSouza, B Mullaney, C McKenna, C Brewer, C Olimpio, C Clabby, C Crosby, C Jenkins, C Armstrong, C Bowles, C Brooks, C Byrne, C Maurer, D Baralle, D Chubb, D Stobo, D Moore, DO Sullivan, D Donnelly, D Randhawa, D Halliday, E Atkinson, E Rauter, E Johnston, E Maher, E Sofianopoulou, E Petrides, F McRonald, F Pelz, I Frayling, G Corbett, G Rea, H Clouston, H Powell, H Williamson, H Carley, HJW Thomas, I Tomlinson, J Cook, J Hoyle, J Tellez, J Whitworth, J Williams, J Murray, J Campbell, J Tolmie, J Field, J Mason, J Burn, J Bruty, J Callaway, J Grant, J Del Rey Jimenez, J Pagan, J VanCampen, J Barwell, K Monahan, K Tatton-Brown, KR Ong, K Murphy, K Andrews, K Mokretar, K Cadoo, K Smith, K Baker, K Brown, K Reay, K McKay Bounford, K Bradshaw, K Russell, K Stone, K Snape, L Crookes, L Reed, L Taggart, L Yarram-Smith, L Cobbold, L Walker, L Walker, L Hawkes, L Busby, L Izatt, L Kiely, L Hughes, L Side, L Sarkies, K-L Greenhalgh, M Shanmugasundaram, M Duff, M Bartlett, M Watson, M Owens, M Bradford, M Huxley, M Slean, M Ryten, M Smith, M Ahmed, N Roberts, C O'Brien, O Middleton, P Tarpey, P Logan, P Dean, P May, P Brace, R Tredwell, R Harrison, R Hart, R Kirk, R Martin, R Nyanhete, R Wright, R Martin, R Davidson, R Cleaver, S Talukdar, S Butler, J Sampson, S Ribeiro, S Dell, S Mackenzie, S Hegarty, S Albaba, S McKee, S Palmer-Smith, S Heggarty, S MacParland, S Greville-Heygate, S Daniels, S Prapa, S Abbs, S Tennant, S Hardy, S MacMahon, T McVeigh, T Foo, T Bedenham, T Cranston, T McDevitt, V Clowes, V Tripathi, V McConnell, N Woodwaer, Y Wallis, Z Kemp, G Mullan, L Pierson, L Rainey, C Joyce, A Timbs, A-M Reuther, B Frugtniet, B DeSouza, C Husher, C Lawn, C Corbett, D Nocera-Jijon, D Reay, E Cross, F Ryan, H Lindsay, J Oliver, J Dring, J Spiers, J Harper, K Ciucias, L Connolly, M Tsang, R Brown, S Shepherd, S Begum, S Daniels, T Tadiso, T Linton-Willoughby, H Heppell, K Sahan, L Worrillow, Z Allen, C Watt, M Hegarty, R Mitchell, R Coles, G Nickless, E Cojocaru, I Doal, F Sava, C McCarthy, R Jeeneea, D Goudie, M McConachie, S Botosneanu, G Kavanaugh, K Russell, C Sherlaw, O Tsoulaki, C Forde, E Petley, A-B Jones, K Oprych, S Pryde, Z Hyder, N Elkhateeb, R Braham, L Hanington, C Huntley, R Irving, A Sadan, M Ramos, C Elliot, D Wren, D Lobo, J McLean, D May, L Kearney, T Campbell, K Asakura, L Alwadi, R O’Shea, J Gabriel, L Chiecchio, P Bowman, LA Sutton, C Walsh, V Cloke, D Ucanok, J Davies, B Pleasance, E Maguire, A Whaite, S Best, S Westbury, A Logan, D Navarajasegaran, A Bench, P Wightman, A Cartwright, E Higgs, J Bott, H Whitehouse, J Stevens, D Martin, J Dunlop, S Thomas, C Sau, S Farndon, N Coleman, P Angelini, M Duff, H Massey, C Rowlands, C Garcia-Petit, K Gillespie, A Alder, E Middleton, C Cassidy, N Orfali, A Webb, A Luharia, N Walker, J Charlton, A Andreou, J Peddie, M Khan, L Wilkinson, H Bezuidenhout, M Edis, A Callard, P Ostrowski, P Moverley, K Bean, A Dunne, A Moleirinho, S Waller, K Cox, L Greensmith, A Brittle, N Gossan, L Freestone, C Shak, T Langford, Y Clinch, H Livesey, S Borland, A Joshi, K Wall, A Whitworth, A Wilsdon, K Edgerley, S Pugh, N Chrysochoidi, S Mutch, C McMullan, Y Johnston, M Muraru, A May, R Begum, C Smith, R Patel, I Bhatnagar, A Taylor, D Brown, J Willan, S Taylor, K Jones, K Cox, C Ramsden, O Taiwo, J Jaudzemaite, R Sharmin, L Young, C O’Dubhshlaine, L McSorley, I Abreu Rodriguez, S Lillis, P Alexopoulos, E Mortensson, L Kingham, R Moore, M Kosicka-Slawinska, S Aslam, R Wells, A Carter, H Warren, E Rolf, H Reed, L Pearce, D Lock, F Ali

**Affiliations:** 1 Division of Genetics and Epidemiology, The Institute of Cancer Research, London, UK; 2 St George's University Hospitals NHS Foundation Trust, London, UK; 3 Sheffield Diagnostic Genetics Service, NEY Genomic Laboratory Hub, Sheffield Children's NHS Foundation Trust, Sheffield, UK; 4 Manchester Centre for Genomic Medicine and NW Laboratory Genetics Hub, Manchester University NHS Foundation Trust, Manchester, UK; 5 The Leeds Genetics Laboratory, NEY Genomic Laboratory Hub, Leeds Teaching Hospitals NHS Trust, Leeds, UK; 6 Wessex Genetics Laboratory Service, University Hospital Southampton NHS Foundation Trust, Salisbury, UK; 7 Genomics and Molecular Medicine Service, Nottingham University Hospitals NHS Trust, Nottingham, UK; 8 Institute of Medical Genetics, Cardiff and Vale University Health Board, University Hospital of Wales, Cardiff, UK; 9 West of Scotland Centre for Genomic Medicine, Queen Elizabeth University Hospital, NHS Greater Glasgow and Clyde, Glasgow, G51 4TF, UK; 10 South East Scotland Clinical Genetics, Western General Hospital, Edinburgh, UK; 11 CHI Crumlin Department of Clinical Genetics, Dublin, Ireland; 12 The Royal Marsden NHS Foundation Trust, London, UK; 13 Peninsula Regional Genetics Service, Royal Devon University Healthcare NHS Foundation Trust, Exeter, UK; 14 Department of Clinical and Biomedical Sciences, University of Exeter Medical School, Exeter, UK; 15 Big Data Institute, University of Oxford, Oxford, UK; 16 Program in Medical and Population Genetics, Eli and Edythe L Broad Institute of Harvard and MIT, Cambridge, Massachusetts, USA

**Keywords:** Genetics, Genetic Variation, Genetics, Population, Genetic Testing

## Abstract

**ABSTRACT:**

**Background:**

The 2015 American College of Medical Genetics/Association of Molecular Pathology (ACMG/AMP) variant classification framework specifies that case-control observations can be scored as ‘strong’ evidence (PS4) towards pathogenicity.

**Methods:**

We developed the PS4-likelihood ratio calculator (PS4-LRCalc) for quantitative evidence assignment based on the observed variant frequencies in cases and controls. Binomial likelihoods are computed for two models, each defined by prespecified OR thresholds. Model 1 represents the hypothesis of association between variant and phenotype (eg, OR≥5) and model 2 represents the hypothesis of non-association (eg, OR≤1).

**Results:**

PS4-LRCalc enables continuous quantitation of evidence for variant classification expressed as a likelihood ratio (LR), which can be log-converted into log LR (evidence points). Using PS4-LRCalc, observed data can be used to quantify evidence towards either pathogenicity or benignity. Variants can also be evaluated against models of different penetrance. The approach is applicable to balanced data sets generated for more common phenotypes and smaller data sets more typical in very rare disease variant evaluation.

**Conclusion:**

PS4-LRCalc enables flexible evidence quantitation on a continuous scale for observed case-control data. The converted LR is amenable to incorporation into the now widely used 2018 updated Bayesian ACMG/AMP framework.

**WHAT IS ALREADY KNOWN ON THIS TOPIC**

- The 2015 American College of Medical Genetics/Association of Molecular Pathology (ACMG/AMP) variant classification framework is a categorical system affording evidence allocation at different levels for various evidence codes.
- However, for the PS4 code, standard application of case-control evidence towards pathogenicity is specified as ’strong’.
- A Bayesian approach to variant classification, by which evidence items are quantified as numerical likelihood ratios (LRs), was proposed in 2018 and has since been widely implemented.

**WHAT THIS STUDY ADDS**

- The PS4-likelihood ratio calculator (PS4-LRCalc) approach enables comparison, based on the observed frequency of a variant in cases and controls, of the underlying likelihood of association (OR≥5 or another prespecified OR) versus the underlying likelihood of non-association (OR≤1), thus enabling generation of a numerical LR reflective of the evidence.

**HOW THIS STUDY MIGHT AFFECT RESEARCH, PRACTICE OR POLICY**

- The PS4-LRCalc approach (and accessible online tool) will enable more accurate and quantitative assimilation of case control data into variant classification, consistent with adoption by ACMG/AMP and ClinGen of the Bayesian LR structure.
- In addition, there is potential for quantitation of case control evidence towards benignity and evaluation of pathogenicity against different penetrance models.

## Introduction

The American College of Medical Genetics/Association of Molecular Pathology (ACMG/AMP) published in 2015 a provisional framework for the interpretation and classification of genomic sequence variants.^1^ Codes and weightings were provided for evidence items comprising the frequency of variant observations in humans with and without phenotype, predictions for sequence changes of protein and splicing impact and assays of variant function. The intention of the 2015 ACMG/AMP framework was to improve the consistency and robustness of variant classifications. Nevertheless, the authors recognised the 2015 framework to be provisional. The ClinGen Sequence Variant Interpretation (SVI) Group has developed modifications and numerical specifications for many evidence codes, with detailed exposition of these relating to individual genes or sets of genes developed by Variant Curation Expert Panels (VCEPs).^2^,^11^

In the original 2015 ACMG/AMP framework, four ordinal evidence weightings were delineated (supporting, moderate, strong or very strong), with specification for how evidence items attaining these weightings were to be combined to provide overall classifications. In 2018, Tavtigian and SVI colleagues proposed a Bayesian reconfiguration of the framework, whereby evidence would instead be quantified as likelihood ratios (LRs, also termed OddsPath, odds of pathogenicity).^12^ By taking the logarithm (to base 2.08), these LRs might be translated into exponent (evidence) points (EPs), in such a way that the previous evidence weightings were transformed into a geometrical progression of supporting (1 EP), moderate (2 EPs), strong (4 EPs) and very strong (8 EPs; table 1).^13 14^ Assuming a prior probability of pathogenicity of 10%, EPs can be summed (or the product of the LRs calculated) and can then be converted to posterior probabilities and assigned to one of five overall variant classifications: benign (<1% probability of pathogenicity), likely benign (1%–10%), variant of uncertain significance (10%–90%), likely pathogenic (90%–99%) and pathogenic (>99%). The SVI and VCEPs have applied this LR-based approach to quantify the evidence weighting for data relating to functional assays (PS3/BS3), phenotype specificity (PP4) and in silico predictions (PP3/BP4).^315^,^18^ It has been advised the forthcoming revision of the 2015 ACMG/AMP framework will adopt the LR-EP system and that non-integer EPs may be permissible, a substantial evolution from the confined prescriptions for evidence combinations laid out in the original 2015 framework.^19^

**Table 1:** Flexible LR-based assignment of evidence weightings, as described in the 2018 Bayesian evolution of the ACMG/AMP framework

LR ratio range	Evidence points	Direction of evidence	ACMG/AMP (2015) evidence strength
≥350.4 (2.08^8^)	8	Towards pathogenicity	Very strong
≥18.72 (2.08^4^) and <350.4	4	Towards pathogenicity	Strong
≥4.33 (2.08^2^) and <18.72	2	Towards pathogenicity	Moderate
≥2.08 (2.08^1^) and <4.33	1	Towards pathogenicity	Supporting
≤0.48 (2.08^−1^) and >0.23	−1	Towards benignity	Supporting
≤0.23 (2.08^−2^) and >0.053	−2	Towards benignity	Moderate
≤0.053 (2.08^−4^) and >0.00285	−4	Towards benignity	Strong
≤0.00285 (2.08^−8^)	−8	Towards benignity	Very strong

PS4-LRCalc was developed in Python (vV.3.11), and analyses were performed using PyCharm (vV.23.1.1, Professional Edition) for remote development on a high-performance computing cluster. The online tool for LR calculator use was developed using Shiny for Python.

As described by Tavtigian *et al,* evidence criteria for which a likelihood ratio towards pathogenicity can be quantified may be converted to EPs through log-transformation (to base 2.08).^13 14^ This continuous approach reflects evidence strength quantitatively, in contrast to the categorical approach of the 2015 ACMG/AMP framework.

ACMG/AMPAmerican College of Medical Genetics/Association of Molecular PathologyEPsexponent pointsLRlikelihood ratioPS4-LRCalcPS4-likelihood ratio calculator

One of the most fundamental observations indicating that a variant is disease-associated (i.e. pathogenic) is observation of that variant at a higher frequency in individuals with the relevant disease/phenotype (cases) than in those without (controls). Such case-control evidence was assigned code PS4 in the 2015 ACMG/AMP framework.^1^ In the 2015 framework paper, discussion by authors highlighted that the strength of association might vary between different gene-phenotype dyads, as well as that the precision of the estimated effect size (i.e. the confidence interval, or CI) was an important consideration alongside the point estimate; the recommendation in the paper was allocation of ‘strong’ evidence for PS4 where OR>5 and the lower 95% CI>1.^1^ However, PS4 is one of the few codes for which there has been no subsequent specification by the SVI, with the stipulations by VCEPs varying widely around how evidence might be allocated for PS4, but largely relating to case-counting rather than case-control association signal.^7 11^ The overlap of use of the same data sets for PS4 as with codes for variant frequency in controls (PM2, BA1, BS1) and lack of provision for case-control data evaluation towards benignity have also been recognised as current limitations.

There is therefore a requirement for a mechanism by which to translate across from the frequentist stipulations relating to case-control ORs (as per the current 2015 ACMG/AMP framework) into a Bayesian quantitation of an LR (commensurate with the 2018 SVI framework evolution and forthcoming ACMG framework revision).^12 13^ Recently, Zanti *et al* (1) analysed individual-level SNP-array data on 75 657 breast cancer cases and 52 987 controls of European ancestry, (2) calculated age-specific log-relative risk from survival analysis and (3) generated LRs for 24 *BRCA1* and 68 *BRCA2* variants.^20^ Such methodology is well suited for comprehensive prospective analyses of large epidemiological case-control data sets where individual-level annotations for age and other parameters are available.

However, we also require tools accessible to clinical diagnostic scientists to empower flexible and accurate quantitation of evidence from summary-level case-control data in the context of reactive classification of clinically identified variants (ie, real-time classifications undertaken by diagnostic clinical scientists). There are several cautions regarding clinical application of case-control data for variant classification (on which we expand further in the discussion). These include (1) Matching for ancestry in cases and controls (2) Caution where there is differential quality control (QC) between cases and controls (3) Caution in accuracy of phenotypes, especially when reliant on small numbers of ‘cases’ and/or literature reports (4) Ascertainment of cases based on enrichment for family history.

**Figure 1:**
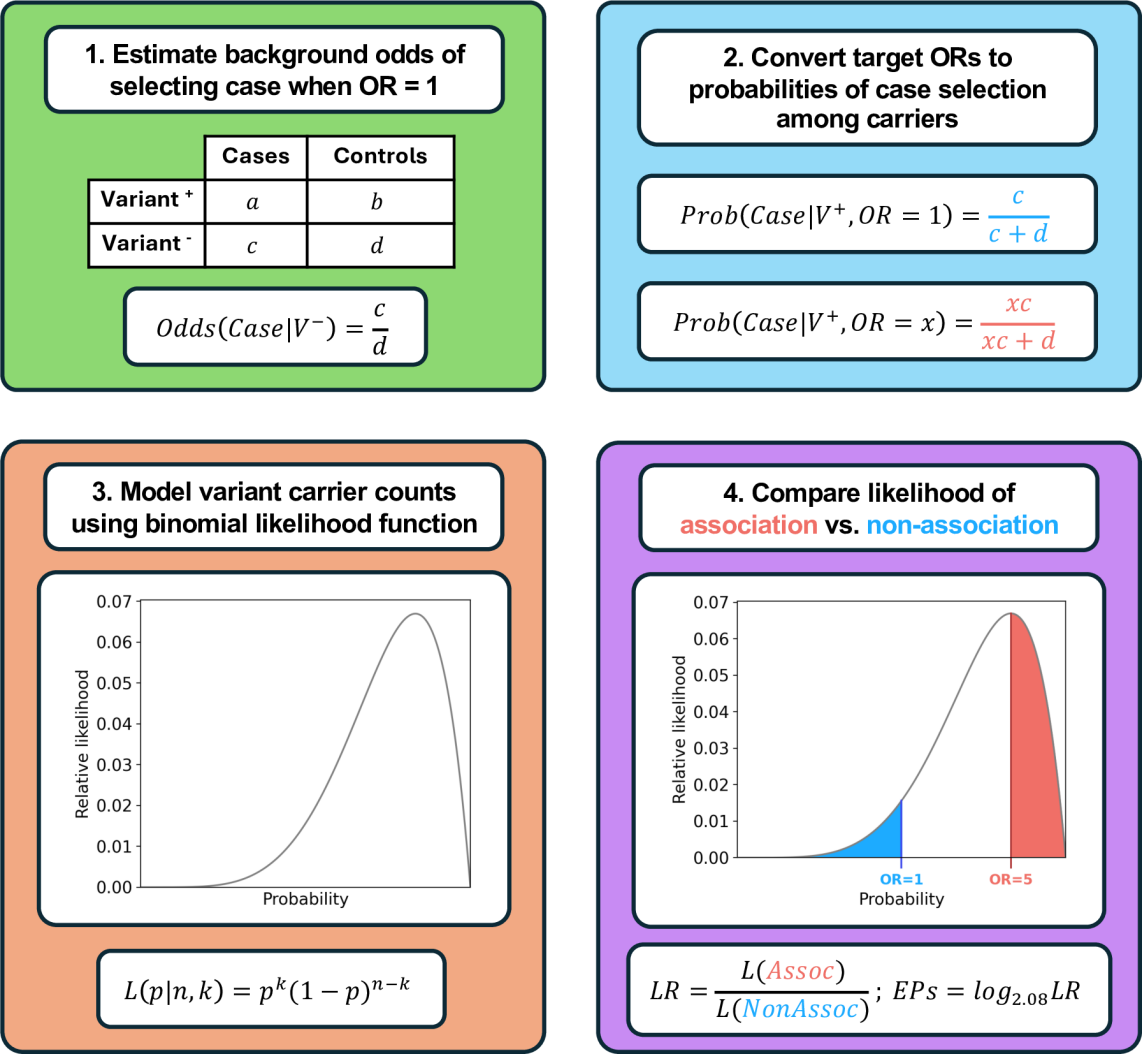
Overview of PS4-LRCalc framework for flexible PS4 application. (1) For a given set of case-control variant observations, the expected background odds of selecting a case among variant carriers are calculated using the equivalent observed odds among non-carriers. (2) The background odds are then scaled according to the ORs of association and non-association and converted to an expected probability of selecting a case among variant carriers under each hypothesis. (3) Variant observations in cases and controls are then modelled using a binomial likelihood function, which evaluates the likelihood that a given probability p of case selection would generate the observed data (k variant observations in cases across n total observations); note that these probabilities directly convert to odds values, which in turn generate a continuum of ORs across all possible values of p. (4) The likelihood ratio (LR) towards pathogenicity is determined by quantifying the total likelihood of association (L(Assoc); red area under curve) and dividing it by the total likelihood of non-association (L(NonAssoc); blue area under curve). The LR can then be converted to Tavtigian exponent points (EPs) by taking its log (to base 2.08). PS4-LRCalc, PS4-likelihood ratio calculator.

We present here the PS4-LR calculator (PS4-LRCalc; figure 1), by which the observed frequency of a given variant in a series of cases can be compared with the observed frequency in a series of controls to quantitatively compare (1) The likelihood of the variant having an effect size at (or above) a specified level (target OR of association) to (2) The likelihood of the variant having an effect size at (or below) a specified level (target OR of non-association). The ratio of these two likelihoods generates an LR that, when converted as described above, provides EPs of the form used within the Bayesian-points-ACMG framework, as described by Tavtigian *et al*.^12 14^ If derived from independently ascertained case-control series, these points can then be summed to generate a combined PS4 Score.

## Methods

### Derivation of LR

We assume that observations of variant counts in cases and controls follow a binomial distribution. We use the binomial likelihood function to compute likelihoods for two models that represent competing hypotheses about the risk of disease associated with a specific variant. The first model (the hypothesis of association) stipulates that the underlying effect size generating the observed variant distribution comprises an OR greater than the stated target OR of association (for example OR≥5). The second model (the hypothesis of non-association) is that the variant is not disease-causing. In the second model, it is assumed that the underlying effect size is less than the stated target OR of non-association, typically OR≤1 (noting that this may also encompass a protective effect; see table 1). The likelihoods of the hypotheses of association and non-association, given the observed data, are hereafter termed the likelihood of association and likelihood of non-association, respectively (see supplementary methods in the online supplemental material for additional detail and worked example).

The PS4-likelihood ratio (PS4-LR) towards pathogenicity (equivalent to the odds of pathogenicity in the Bayesian framework described by Tavtigian *et al*) is calculated by dividing the likelihood of association by the likelihood of non-association.^12 13^ The LR is then converted to a logarithm (of base 2.08) to generate a log likelihood ratio (LLR), also termed EPs, which correspond to PS4 evidence weighting in the 2015 ACMG/AMP framework, as shown in table 1.

## Results

### Quantifying evidence towards pathogenicity: large, balanced case-control data sets (cancer susceptibility genetics scenario)

**Figure 2:**
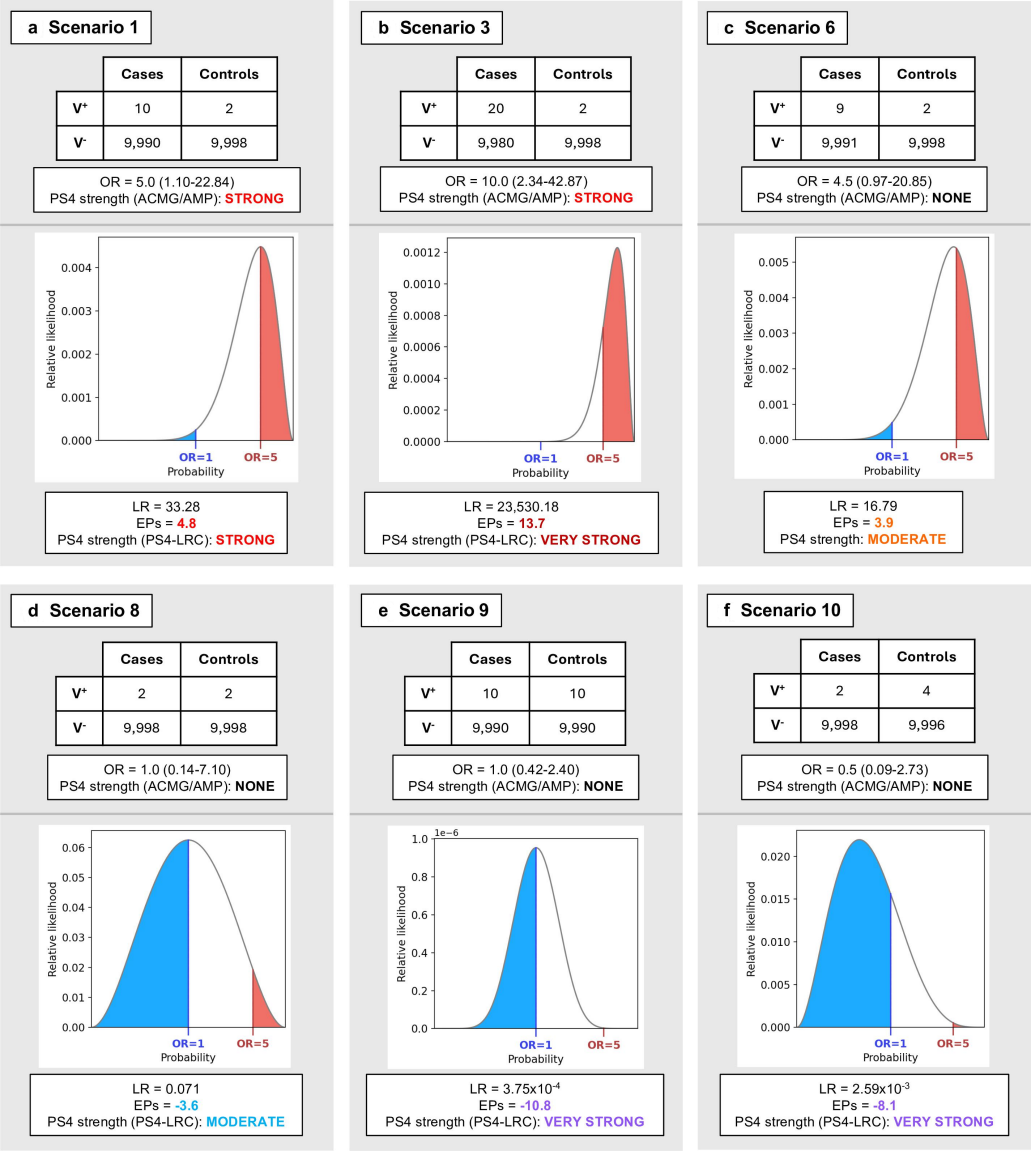
Comparison of applicable strength for the PS4 criterion between the 2015 ACMG/AMP guidelines and PS4-LRCalc for selected case-control scenarios. Counts of carriers (V^+^) and non-carriers (V^−^) of a variant between cases and controls are illustrated for exemplar scenarios indicative of (**A–C**) pathogenicity and (**D–F**) benignity shown in tables2  3. In the PS4-LRCalc approach, the distribution of variant carrier observations between cases and controls is modelled using a binomial likelihood curve, in which the likelihood of association (OR≥5, red) is divided by the likelihood of non-association (OR≤1, blue) to generate a likelihood ratio (LR) towards pathogenicity, that can then be converted to Tavtigian exponent points (EPs) towards pathogenicity or benignity and a corresponding applicable evidence strength under the 2015 ACMG/AMP framework. Notably, our approach allows assignation of EPs for scenarios that may not fulfil the existing ACMG/AMP PS4 guidance, including in support of benignity. ACMG/AMP, American College of Medical Genetics/Association of Molecular Pathology; PS4-LRCalc, PS4-likelihood ratio calculator.

In tables2  3 and figure 2A–F, we present illustrative scenarios of hypothetical variants observed in 10 000 cases and 10 000 controls, applying for our hypothesis of association a target OR of 5. This OR was selected for demonstration on account of being the threshold for disease association proposed in the 2015 ACMG/AMP framework. Thus, in each case an LR is generated from comparison of the likelihood of the true underlying OR being ≥5 against the likelihood of the true underlying OR being ≤1 (the target OR of non-association). In scenarios 1–3 (table 2), ‘strong’ evidence would have been awarded in the existing 2015 ACMG/AMP framework PS4 specification (2015-ACMG-PS4), as the observed OR exceeds 5 and lower 95% CI exceeds 1. However, the magnitude and confidence of association represented by these three scenarios of observed data vary widely: the LRs vary 10^11^-fold and EPs range from 4.8 to 43.0. Notably, in scenario 1, in which the OR=5 and strength of association lies just within prestated statistical significance (p<0.05), the LR is 33.3, equating to allocation of EPs just above the threshold of 4 required for strong evidence.

**Table 2:** Exemplar case-control scenarios generating exponent points (EPs) towards pathogenicity when using PS4-LRCalc

Scenario number	Case number with variant/total cases	Control number with variant/total controls	Observed effect size		ACMG 2015 Framework (OR≥5, LCI≥1)	Likelihood of OR≥5 versus likelihood of OR≤1	
			OR (95% CI)	p-value (Fisher’s exact test)		LR	EPs
1	10/10 000	2/10 000	5.0 (1.10 to 22.84)	0.04	Strong	33.28	4.8
2	100/10 000	20/10 000	5.0 (3.12 to 8.15)	4.69×10^–14^	Strong	3.97×10^13^	42.8
3	20/10 000	2/10 000	10.0 (2.34 to 42.87)	1.20×10^–4^	Strong	2.35×10^4^	13.7
4	5/10 000	1/10 000	5.0 (0.58 to 42.82)	0.22	None/?	5.29	2.3
5	4/10 000	1/10 000	4.0 (0.45 to 35.80)	0.37	None/?	2.41	1.2
6	9/10 000	2/10 000	4.5 (0.97 to 20.85)	0.07	None/?	16.79	3.9

Shown are illustrative sets of variant observations in case and control data sets of equal size (10 000 individuals each) and the respective likelihood ratio (LR) and exponent points (EPs) generated for each using PS4-LRCalc. In scenarios 1–3, assignation of PS4 at ‘strong’ would have been possible under the 2015 ACMG/AMP framework; integration of PS4-LRCalc allows more refined quantification of evidence strength, such that the equivalent of ‘very strong’ evidence can be applied for scenarios 2 and 3. The variants depicted in scenarios 4–6 fail to fulfil the 2015 ACMG/AMP criteria of OR≥5 and p<0.05 for application of PS4; thus any evidence allocation would be on the basis of professional judgement. Scenarios 1, 3 and 6 are further illustrated in figure 2A–C.

ACMGAmerican College of Medical GeneticsAMPAssociation of Molecular PathologyEPexponent pointLRlikelihood ratioORodds ratioPS4-LRCalcPS4-likelihood ratio calculator

**Table 3:** Exemplar case-control scenarios generating exponent points (EPs) towards benignity when using PS4-LRCalc

Scenario number	Case number with variant/total cases	Control number with variant/total controls	Observed effect size		ACMG 2015 Framework (OR≥5, LCI≥1)	Likelihood of OR≥5 versus likelihood of OR≤1	
			OR (95% CI)	p-value (Fisher’s exact test)		LR	EPs
7	1/10 000	1/10 000	1.0 (0.06 to 15.99)	1.00	None	0.15	−2.6
8	2/10 000	2/10 000	1.0 (0.14 to 7.10)	1.00	None	7.10×10^–2^	−3.6
9	10/10 000	10/10 000	1.0 (0.42 to 2.40)	1.00	None	3.75×10^–4^	−10.8
10	2/10 000	4/10 000	0.5 (0.09 to 2.73)	0.69	None	2.59×10^–3^	−8.1
11	2/10 000	10/10 000	0.2 (0.04 to 0.91)	0.04	None	1.55×10^–7^	−21.4

Shown are illustrative sets of case-control observations in data sets of equivalent size and their equivalent likelihood ratios (LRs) and exponent points (EPs) under the PS4-LRCalc model. The 2015 ACMG/AMP framework does not permit the use of lack of case-control signal as evidence for benignity. However, in scenarios 7–9, calculation of LRs using PS4-LRCalc allows application of PS4 in the benign direction at increasing strength—from the equivalent of moderate to very strong—as the number of variant observations increases. In scenarios 10 and 11, EPs for variants with ostensibly protective effects, that is, observed at higher frequency in controls than cases, reach the equivalent of ‘very strong’ in the benign direction. Scenarios 8, 9 and 10 are further illustrated in figure 2D–F. LCI, lower confidence interval; OR, odds ratio.

ACMGAmerican College of Medical GeneticsAMPAssociation of Molecular PathologyEPexponent pointLRlikelihood ratioORodds ratioPS4-LRCalcPS4-likelihood ratio calculator

Conversely, in scenarios 4–6 (table 2), based on OR≥5 with p<0.05, no evidence would have been allocated under 2015-ACMG-PS4. However, in scenario 4, based on the observed data, the likelihood of association is more than fivefold greater than the likelihood of non-association: this equates to 2.3 EPs. In scenario 5, one fewer instance of the variant was observed in the case series compared with scenario 4, meaning the observed OR is lower (OR=4.0; 95% CI 0.45 to 35.80) but nevertheless, the CI readily encompasses the target OR of association (OR=5), and the likelihood of association (that the true underlying is OR≥5) is more than twofold greater than the likelihood of non-association (that the true underlying OR≤1), translating to 1.2 EPs. Similarly, in scenario 6 (figure 2C), the observed OR is 4.5 (0.97–20.84): while no evidence would be allocated under 2015-ACMG-PS4 based on OR≥5 with p<0.05, the likelihood of association (OR≥5) is almost 17-fold greater than the likelihood of non-association (OR≤1), which equates to 3.9 EPs.

### Quantifying evidence towards benignity

PS4-LRCalc also enables quantitation of evidence towards benignity, as illustrated in table 3. Again, we considered in each case the likelihood of association (target OR=5) versus the likelihood of non-association (target OR=1) based on observed data. In scenarios 7–9, we illustrate a range of scenarios in which the observed OR is OR=1, but with increasing numbers of variant observations there is increasingly stronger evidence provided towards benignity. In scenarios 10 and 11, the frequency of the variant in controls is greater than that in cases, providing increasingly powerful evidence towards benignity.

### Quantifying evidence towards pathogenicity: different models of penetrance

In clinical cancer susceptibility genetics, genes associated with breast cancer are deemed to be of high penetrance if the association between pathogenic variants in that gene and phenotype is typically of OR≥4 (eg, *BRCA1*, *BRCA2*), while genes for which pathogenic variants are typically of effect size (OR) 2–4 are deemed to be of moderate penetrance (eg, *CHEK2*). However, for some variants in *BRCA1* and *BRCA2*,^21^ observed data suggest reduced penetrance for breast cancer of OR=2–4, risks more comparable to those ascribed to moderate penetrance genes.^22^ Guidance exists for clinical management of patients with these reduced penetrance *BRCA1*/*BRCA2* variants.^21^ It may therefore be of utility on occasion to be able to assess *BRCA1*/*BRCA2* case-control variant data against both models of penetrance: considering first evidence of association at standard high penetrance (OR≥4) and subsequently for evidence of association at reduced penetrance (OR≥2). For example, if a variant is observed at a frequency of 12/10 000 in cases and 6/10 000 in controls, these observations would not constitute evidence for association against a target OR of association of 4, while against a target OR of 2 these observations would constitute moderate evidence (LR=5.5, EP=2.3; see online supplemental table 1).

### Quantifying evidence towards pathogenicity: unbalanced data sets with small case series (rare disease genetics scenario)

We present in table 4 illustrative ultra-rare disease-type scenarios; that is, small numbers of variant observations in modest-sized case series being compared with large population control cohorts. We illustrate the impact of varying the hypothesis of association, considering OR≥10, OR≥100 and OR≥1000 in keeping with effect sizes commensurate with very rare Mendelian diseases (table 4 and online supplemental table 2). While the LRs and EPs are numerically accurate on the basis of correct variant observations and denominators for cases and controls, particularly critical in these scenarios is consideration of ancestry, accuracy of genotyping and phenotyping, and a correct denominator for the case series (see Discussion).

**Table 4:** Exemplar case-control rare disease-type scenarios

Scenario number	Case number with variant/total cases	Control number with variant/total controls	Observed effect size		Likelihood OR≥10 versus OR≤1		Likelihood OR≥1000 versus OR≤1	
			OR (95% CI)	p-value (Fisher’s exact test)	LR	EPs	LR	EPs
12	2/30	2/300 000	10 700 (1458 to 78 746)	5.80×10^–8^	1.23×10^11^	34.9	1.22×10^11^	34.9
13	5/30	20/300 000	3000 (1044 to 8619)	3.73×10^–16^	1.30×10^19^	60.1	1.28×10^19^	60.1
14	2/300	20/300 000	101 (23 to 433)	2.27×10^–4^	5.86×10^5^	18.1	20.63	4.1
15	5/300	20/300 000	254 (95 to 682)	5.03×10^–11^	4.91×10^12^	39.9	7.03×10^9^	31.0
16	5/300	50/300 000	102 (40 to 257)	3.21×10^–9^	3.57×10^10^	33.2	3.1	1.5
17	3/30	15/15 000	111 (30 to 406)	5.74×10^–6^	2.52×10^7^	23.3	497.1	8.5
18	3/30	30/15 000	55 (16 to 193)	3.76×10^–5^	2.14×10^6^	19.9	4.75×10^–5^	−13.6

In the seven scenarios shown here, as may be typical in a rare-disease clinical setting, the frequency of variants in small-to-modest case series is evaluated against variant frequencies as might be derived from population-scale data sets, using appropriate ancestry-matched subsets therein. All scenarios would attain PS4 under the 2015 ACMG/AMP specification of OR>5 and p<0.05. We apply PS4-LRCalc under hypotheses of association of OR≥10 and OR≥1000, the latter arguably being more consistent with very rare disease. We illustrate in scenarios 14,16 and 18 the substantial impact of differing this target OR against which observed data are evaluated. These use cases assume fully robust genotyping and phenotyping: sensitivity assessment and adjustment may be warranted in scenarios where numbers are small and/or there is uncertainty regarding the robustness of data quality/ascertainment (eg, in scenarios 12 and 14 in which there are only two case observations). ORs and associated 95% CIs are rounded to the nearest whole number here for ease of visualisation; full data are displayed in online supplemental file 1).

ACMG/AMPAmerican College of Medical Genetics/Association of Molecular PathologyEPexponent pointLRlikelihood ratioORodds ratioPS4-LRCalcPS4-likelihood ratio calculator

### Approaches to accommodate data uncertainty

The PS4-LRCalc approach provides, *based on the observed data*, quantitation of the comparative likelihoods of the underlying OR being at or above a higher value (target OR of association) versus at or below another, lower value (target OR of non-association). PS4-LRCalc will inherently reflect sample size (power, sampling variability), namely that the magnitude of the LR attained for a variant of a given frequency and strength of disease association will be determined by the magnitude of the case and control data series. If the observed data are accurate and robust, then the outputted LR most directly quantifies the evidence towards pathogenicity (or benignity) afforded from the observed data.

However, on occasion there may be uncertainty regarding the accuracy of genotyping, phenotyping, ancestry or denominator of (in particular) the case series. Notably, this is an issue inherent to any application of case (or control) data towards variant classification, rather than the issue being particular to the PS4-LRCalc.

By virtue of its parameterisation, PS4-LRCalc affords various options for introducing caution (often also termed ‘conservatism’) by which the output metric for case-control analysis will be accordingly dampened (attenuated). The selection and degree of dampening should be predicated on the level of uncertainty of data accuracy and context in which outputs are being applied (reciprocal approaches could be applied to dampen/attenuate the allocation of evidence towards benignity but this is less typically a use case of concern):

1. Adjustment of the opposing hypothesis: Quantitation of evidence towards pathogenicity can be attenuated by adjusting the competing hypothesis. That is, where unadjusted data provide evidence towards pathogenicity, the target OR of non-association may be increased. In online supplemental table 3, we illustrate how the LR/LLR are impacted by comparing a target OR of association of OR=5 to different target ORs assigned as representing non-association, namely OR≤1 (default), OR≤2 and OR≤5.
2. Application of a CI to the target ORs (the target OR of association and the target OR of non-association): Rather than using the target OR for association (eg, OR=5), it is possible to use the lower (70%, 90% or 95%) CI of this OR estimate, as derived from the expected counts under the hypothesis of association. Rather than using the target OR for non-association (eg, OR=1), it is possible to use the upper (70%, 90% or 95%) CI of this estimate, as derived from the expected counts under the hypothesis of non-association (see supplementary methods in the online supplemental material). An LR incorporating one or both of these values is thus attenuated (ie, dampened) compared with an LR derived using the direct target ORs. Of note, where there are low total variant observations and/or unbalanced case-control data set size this will have a substantive impact on the standard error of the OR, such that addition of a CI will result in accordingly aggressive diminution of the LR (ie, this approach may be highly punitive in these scenarios) (online supplemental table 4).
3. Sensitivity analysis: By reducing the number of case observations and/or increasing control observations (commensurate with degree of uncertainty and ‘trust’ in the data), it is possible to assess the robustness of the unadjusted case-control signal. For example, if the count of case observations with the variant is n=2, reducing the case count to n=1 and reconducting LR quantification may inform the confidence around the original prediction. This strategy may be particularly pertinent in rare disease scenarios in which the case denominator is modest, meaning that each observation of an instance of the variant in a case is contributing substantially to the evidence weighting; this approach should provide reasonable mitigation against biased ascertainment, erroneous phenotyping or winner’s curse.
  The logical extension of the sensitivity analysis principle is that for the n=1 case variant scenario, application of PS4 is disallowed (this being equivalent to n_case_=1 being sensitivity-tested as n_case_=0).

There are two additional ‘edge-case’ restrictions we have identified pertaining to unusual comparisons of likelihoods. The first scenario involves caution where the LR is generated from comparison of two hypotheses each of minuscule likelihood. In scenarios with a well-powered case-control signal for a variant with an observed OR of intermediate value (eg, OR=2.5), the target ORs of non-association (eg, OR≤1) and association (eg, OR=5) may lie below and above the CI limits of the observed OR estimate, respectively. In these scenarios, the likelihoods of association (at OR≥5) or non-association (at OR≤1) will constitute only a minuscule proportion of the total likelihood space (ie, total area under the curve). The true underlying OR is very highly likely to be of intermediate value and thus to lie confidently in between the two hypotheses. However, if the minuscule likelihood of the observed data hypothesis of association is substantially greater than the minuscule likelihood for the hypothesis of non-association, an LR of sizeable magnitude can be generated (online supplemental figure 1). In such a scenario in practice, there is a priori high confidence of an effect intermediate between the two hypotheses, meaning that interrogation against a much higher target OR of association is thus unlikely to be a clinically meaningful endeavour.

The second scenario involves comparison between zero case observations of the variant (within a small case series) against small numbers of variant observations (in a dramatically larger control series). In this scenario, the extremely wide CI for the estimate for the true case frequency can generate unstable estimates for the LR. Again, this scenario is unlikely to be relevant to current clinical practice but may pertain to bulk processing of variant data sets for assignment of evidence towards benignity.

### Accessible applet for direct access to PS4-LRCalc tool

An online Shiny for Python tool is available at https://turnbull-lab.shinyapps.io/ps4_lrcalc/. This allows the input of (1) Case and control variant observations and denominators, (2) A target OR of association, (3) A target OR of non-association, and (4) (optional) CIs. The outputs include the relative likelihoods, an LR, EPs, a distribution curve of likelihoods and a quantitation of likelihood space occupancy. For results relating to edge-case data scenarios, a ‘warning flag’ is returned highlighting that caution is required regarding the current comparison of the likelihoods scenario, namely (1) where results have low likelihood space occupancy and (2) where there are unbalanced data sets with zero case observations (see above). We also flag requirement for caution where there is a single variant observation in cases (and that scrutiny of phenotypical/genotypical accuracy are essential (see below)).

## Discussion

We present PS4-LRCalc, which enables quantification of evidence towards variant pathogenicity/benignity based on a continuous output from a statistical model. The PS4-LRCalc approach allows evaluation of the observed data against a prespecified ‘target OR of association’ and a prespecified ‘target OR of non-association’, thus quantified as an LR (which effectively serves to bridge the frequentist-Bayesian divide). This PS4-LRCalc approach is applicable across a spectrum of use cases, including both the highly penetrant effects observed from small case series in investigation of ultra-rare Mendelian disease, as well as less penetrant effects inferred from the larger case series available for the investigation of more common phenotypes, for example, for variants in cancer susceptibility genes. The PS4-LRCalc approach is currently only configured for autosomal dominant inheritance (heterozygous variants).

The advantages of the PS4-LRCalc approach include:

- First, the evidence is quantified as an LR, which is then converted into an LLR, which equates to a number of EPs. This approach is consistent with the 2018 Tavtigian-SVI adaptation of the 2015 ACMG framework which is to be adopted in the forthcoming ACMG framework update.
- Second, the evidence strength (LLR) is quantified on a continuous scale, affording direct quantitative reflection of the magnitude of evidence towards pathogenicity afforded by the observed data. This offers dramatically improved flexibility compared with the 2015-ACMG-PS4 standard specification of case-control evidence as strong where OR≥5 and p<0.05.
- Third, the parameterisation of the PS4-LRCalc model allows specification of target ORs of interest.
  - For analysis examining a *BRCA2* variant in unselected breast cancer cases versus controls, a target OR≥4 is appropriate.
  - For ovarian cancer case control data, a target OR≥10 is appropriate.
  - Where cases are highly selected based on family history of hereditary breast-ovarian cancer, a commensurately higher target OR should be applied (see below regarding enrichment factor)
  - Models of reduced penetrance can be explored by setting a lower target OR than is standard for that gene.
- Fourth, the PS4-LRCalc approach can accommodate zero counts in the 2×2 frequency table (a frequent occurrence for variant counts in control series). This obviates application of a Haldane-Anscombe correction with the consequent clumsy and rather arbitrary down-adjustment of the association effect.
- Fifth, this approach readily facilitates more accurate combination of evidence from multiple (independent) case-control studies, with summing of EPs (or multiplication of LRs).
- Sixth, this approach allows quantitation of evidence towards benignity based on observed data; arguably an elegant complement or alternative to the current BA1/BS1 codes, by which evidence towards benignity is assessed based purely on variant frequency in controls.
- Finally, we outline several potential approaches by which to manage uncertainty inherent to clinical data of uncertain quality/provenance.

Application of summary-level case-control data for variant interpretation carries a number of cautions and caveats: these apply equally to the existing 2015-ACMG-PS4 as to the PS4-LRCalc approach:

- There is no presumption in case-control analysis regarding which component of the phenotype is assessed (eg, breast cancer vs ovarian cancer), as long as all cases analysed (those with and without the variant in question) have been collected under the same case/phenotype definition.
- There is no underlying presumption regarding locus heterogeneity: What is being analysed is the frequency of a given variant (thus necessarily from a single gene) in cases versus the frequency of that variant in controls, matched for ancestry and with high genotype quality metrics at the site of variation.
- There should be appropriate matching of ancestry between case and control series.
- The standards for sequencing/genotyping and downstream QC must be considered, in particular where they differ between cases and controls. QC metrics for variant observations in publicly available population data sets should be actively reviewed, for example, examination for gnomAD variants of genotype quality metrics and frequency comparison between exome and genome sources.^23^
- The provenance of the data (eg, if extracted from a publication) is particularly important where variant numbers are small (meaning that single observations may substantially influence the outcome).
- Any metric for case-control association requires accurate denominators. If there is uncertainty regarding the denominator relating to the observed cases, case-control analysis (including PS4-LRCalc) should not be undertaken. Instead, where disease phenotype is extremely rare and distinctive, a ‘case-counting’ approach should be employed using evidence allocation as specified by the respective disease-expert VCEP.
- If there is any level of enrichment or overselection among the case series, this will cause inflation in the observed OR in relation to that of an unselected cohort. If the cohorts have been genotyped for a specific variant of known effect size, an ‘enrichment factor’ for the cohort could be calculated, by which observed ORs may be suitably down-adjusted.
- Using summary-level variant frequencies in cases and controls will disregard differential variant distribution in the context of variable age-related penetrance (namely, where the variant is disproportionately frequent in younger age groups, in which background rates of disease are lower).
- Finally, in the 2015 ACMG/AMP framework, case-control evidence allocation for PS4 was capped at strong and it was mandated that classification as benign/likely benign/likely pathogenic/pathogenic required representation from multiple evidence categories. Use of EPs generated from PS4-LRCalc will need to be compliant with forthcoming stipulations from ClinGen/ACMG/AMP regarding (1) Caps for individual evidence codes. (2) Requirement for evidence from multiple evidence codes. (3) Concomitant use of evidence codes referencing identical source data (namely PM2 and, if case-control data towards benignity is permitted, of BA1 and BS1).

We have created an accessible methodology and user-friendly publicly available tool enabling flexible, accurate quantitation for variant classification of case-control data as an LR and respective EPs. In particular, this approach affords (1) Allocation of lower levels of contributary evidence in instances for which the confidence and/or effect size would not attain the requirements for ‘strong’ currently specified for 2015-ACMG-PS4, (2) Allocation of higher than ‘strong’ levels of contributary evidence where supported by observed data, and (3) Evidence towards benignity. We anticipate this type of approach and tool will be of utility to diagnostic clinical scientists and clinicians with increased availability of newly collated large diagnostic testing and population sequencing data sets, in particular on update of the ACMG/AMP framework, in which evidence quantitation will be more continuous and use an LR/LLR framework.

## supplementary material

10.1136/jmg-2024-110034 online supplemental file 1

## Data Availability

All data relevant to the study are included in the article or uploaded as supplementary information.

## References

[1] Richards S, Aziz N, Bale S (2015). Standards and guidelines for the interpretation of sequence variants: a joint consensus recommendation of the American College of Medical Genetics and Genomics and the Association for Molecular Pathology. Genet Med.

[2] ClinGen (2019). SVI recommendation for in trans criterion (pm3) - version 1.0.

[3] Brnich SE, Abou Tayoun AN, Couch FJ (2019). Recommendations for application of the functional evidence PS3/BS3 criterion using the ACMG/AMP sequence variant interpretation framework. *Genome Med*.

[4] Abou Tayoun AN, Pesaran T, DiStefano MT (2018). Recommendations for interpreting the loss of function PVS1 ACMG/AMP variant criterion. Hum Mutat.

[5] ClinGen (2018). ClinGen sequence variant interpretation recommendation for de novo criteria (ps2/pm6) – version 1.1.

[6] Patel MJ, DiStefano MT, Oza AM (2021). Disease-specific ACMG/AMP guidelines improve sequence variant interpretation for hearing loss. *Genet Med*.

[7] Fortuno C, Lee K, Olivier M (2021). Specifications of the ACMG/AMP variant interpretation guidelines for germline TP53 variants. Hum Mutat.

[8] Kanavy DM, McNulty SM, Jairath MK (2019). Comparative analysis of functional assay evidence use by ClinGen Variant Curation Expert Panels. Genome Med.

[9] Rivera-Muñoz EA, Milko LV, Harrison SM (2018). ClinGen Variant Curation Expert Panel experiences and standardized processes for disease and gene-level specification of the ACMG/AMP guidelines for sequence variant interpretation. Hum Mutat.

[10] Zastrow DB, Baudet H, Shen W (2018). Unique aspects of sequence variant interpretation for inborn errors of metabolism (IEM): The ClinGen IEM Working Group and the Phenylalanine Hydroxylase Gene. Hum Mutat.

[11] Lee K, Krempely K, Roberts ME (2018). Specifications of the ACMG/AMP variant curation guidelines for the analysis of germline CDH1 sequence variants. Hum Mutat.

[12] Tavtigian SV, Greenblatt MS, Harrison SM (2018). Modeling the ACMG/AMP variant classification guidelines as a Bayesian classification framework. *Genet Med*.

[13] Tavtigian SV, Harrison SM, Boucher KM (2020). Fitting a naturally scaled point system to the ACMG/AMP variant classification guidelines. Hum Mutat.

[14] Garrett A, Durkie M, Callaway A (2021). Combining evidence for and against pathogenicity for variants in cancer susceptibility genes: CanVIG-UK consensus recommendations. J Med Genet.

[15] Pejaver V, Byrne AB, Feng B-J (2022). Calibration of computational tools for missense variant pathogenicity classification and ClinGen recommendations for PP3/BP4 criteria. Am J Hum Genet.

[16] Loong L, Cubuk C, Choi S (2022). Quantifying prediction of pathogenicity for within-codon concordance (PM5) using 7541 functional classifications of BRCA1 and MSH2 missense variants. Genet Med.

[17] Garrett A, Loveday C, King L (2022). Quantifying evidence toward pathogenicity for rare phenotypes: the case of succinate dehydrogenase genes, SDHB and SDHD. Genet Med.

[18] Cubuk C, Garrett A, Choi S (2021). Clinical likelihood ratios and balanced accuracy for 44 in silico tools against multiple large-scale functional assays of cancer susceptibility genes. *Genet Med*.

[19] Harrison S (2023). Draft ACMG/AMP/CAP/ClinGen Standards for Sequence Variant Classification; Overview and Timeline for Implementation.

[20] Zanti M, O’Mahony DG, Parsons MT (2023). A likelihood ratio approach for utilizing case-control data in the clinical classification of rare sequence variants: application to *BRCA1* and *BRCA2*. Hum Mutat.

[21] Moghadasi S, Meeks HD, Vreeswijk MP (2018). The *BRCA1* c. 5096G>A p.Arg1699Gln (R1699Q) intermediate risk variant: breast and ovarian cancer risk estimation and recommendations for clinical management from the ENIGMA consortium. J Med Genet.

[22] Easton DF, Pharoah PDP, Antoniou AC (2015). Gene-panel sequencing and the prediction of breast-cancer risk. N Engl J Med.

[23] (2024). GnomAD browser: what do the flags on the browser mean?. https://gnomad.broadinstitute.org/help/what-do-the-flags-on-the-browser-mean.

